# Supplementary material for: Disclosing political partisanship polarizes first impressions of faces
Source: PLoS One. 2022 Nov 9;17(11):e0276400. doi: 10.1371/journal.pone.0276400 (PMC9645606; doi:10.1371/journal.pone.0276400)
Supplement: S1 File — (DOCX) [file pone.0276400.s001.docx]

**Disclosing Political Partisanship Polarizes First Impressions of Faces**

Brittany S. Cassidy, Colleen Hughes, Anne C. Krendl

The information provided below describes the file structure on OSF and reports on additional measures and/or analyses associated with this manuscript.

**OSF File Structure**

There are three folders corresponding to the three experiments reported in the manuscript. Each folder contains in the main directory: a R analysis script describing the data cleaning, analyses, and figure creation; two .rds files for the raw and scored task data (e.g., “Exp1_data.rds”); and two .rds files for the raw and scored behavioral data (e.g., measures of political ideology, demographics; “Exp1_behavioralData.rds”). There is also a results directory containing descriptive and correlational output for the reported tables here and in the manuscript, and the results figures as a .tiff and .jpeg. Experiment 3’s main directory also contains a .xlsx file called “QuestionnaireScoring.xlsx” with participants’ responses to the greater number of questionnaires described below. Detailed comments can be found in the analysis files.

**Experiment 1**

Of 180 participants, 52 identified as Republican, 70 as Democrat, and 57 as undecided. One did not respond. An ANOVA confirmed that political party membership related to composite political ideology, *F*(2, 176) = 83.60, *p* < .001, *η_p_^2^* = .49. Republicans (*M* = 6.55, *SD* = 1.18) were more conservative than undecideds (*M* = 5.03, *SD* = 1.22), *t*(107) = 6.59, *p* < .001, *d* = 1.26, 95% CI [.88, 1.74], and Democrats (*M* = 3.31, *SD* = 1.62), *t*(119.97) = 12.81, *p* < .001, *d* = 2.29, 95% CI [1.79, 3.00]. Undecideds were more conservative than Democrats, *t*(124.36) = 6.84, *p* < .001, *d* = 1.20, 95% CI [.76. 1.76]. Participants identifying as Democrat, Republican, and undecided were unbalanced across Task Version, *χ^2^*(2)=6.83, *p*=.03. Across task versions, participants identifying with each political party had similar ideology (two-way interaction), *F*(2,173)=.42, *p*=.66.

***Exploratory Measures***

Because the extent to which people are affiliated with partisan groups drives their political beliefs and evaluations (e.g., Van Bavel & Pereira, 2018), we characterized partisan affiliations by having people rate their affiliation (“How strongly do you feel affiliated with these political categories?”) with Republicans, Democrats, and undecided groups using scales ranging from 1 *(not affiliated at all)* to 9 *(strongly affiliated)*. Participants also indicated how well they knew someone who was Republican, Democrat, or undecided on a scale from 1 *[not at all]* to 7 *[very much]* and had the choice to indicate that they did not know anyone who affiliated with these groups.

***Supplemental Analyses***

Supplemental Table 1 provides descriptive statistics and intercorrelations supporting the relationships between political ideology and political affiliation with and perceived threat of Republicans, Democrats, and undecideds. Table 5 in the manuscript shows the model results supporting the conclusion that political ideology was related to differential threat perceptions of Republicans and Democrats versus undecideds. Supplemental Table 2 provides further support for this conclusion. Specifically, it shows the overall model results when using Republicans as the reference group thereby allowing a direct comparison of threat perceptions of Republicans versus Democrats (and undecideds). Simple effects from this model are described in the manuscript. Descriptive statistics and intercorrelations between political ideology and partisan experience are reported in Supplemental Table 3.

**Experiment 2**

Thirty-eight participants identified as Republican, 29 as Democrat, and 27 as undecided. An ANOVA verified political party membership corresponded with composite political ideology, *F*(2, 91) = 51.13, *p* < .001, *η_p_^2^* = .53. Republicans (*M* = 6.43, *SD* = 1.01) were more conservative than undecideds (*M* = 4.87, *SD* = 1.13), *t*(63) = 5.86, *p* < .001, *d* = 1.47, 95% CI [1.00, 2.10]; and Democrats (*M* = 3.35, *SD* = 1.57), *t*(45.03) = 9.22, *p* < .001, *d* = 2.34, 95% CI [1.64, 3.47]. Undecideds were more conservative than Democrats, *t*(54) = 4.12, *p* < .001, *d* = 1.11, 95% CI [.56, 1.88]. An ANOVA on composite political ideology using self-identified political party (Republican, Democrat, undecided) and Experiment (1, 2) did not reveal an Experiment effect, *F*(1,267)=.19, *p*=.67; or an interaction, *F*(2,267)=.13, *p*=.88.

**Exploratory Measures**

The same exploratory measures of partisan experience in Experiment 1 were collected in Experiment 2. Their descriptive statistics and intercorrelations are reported in Supplemental Tables 4 and 5.

***Supplemental Analyses***

In the manuscript, Table 5 shows the results of the overall model supporting that impressions of Republicans, Democrats, and undecideds were modulated after Label disclosure by political ideology. Supplemental Table 2 further supports this conclusion by showing the overall model using Republicans (vs. undecideds in Table 5) as the reference group thereby allowing direct comparison of Republicans and Democrats. Simple effects from this model are reported in the manuscript. Supplemental Table 6 replicates the main task analysis reported in the manuscript (Table 6) but using Republicans as the reference group for direct comparison to Democrats. Simple effects from this model are reported in the manuscript.

Supplemental Table 1. *Means (M), standard deviations (SD), and intercorrelations (r) between political ideology, perceived threat, and political affiliation in Experiment 1.*

| Measure | *M* | *SD* | 1 | 2 | 3 | 4 | 5 | 6 | 7 |
| --- | --- | --- | --- | --- | --- | --- | --- | --- | --- |
| 1. Perceiver political ideology | 4.79 | 1.91 | -- |  |  |  |  |  |  |
| 2. Republican threat | 3.31 | 1.73 | -.50**  [-.60, -.38] | -- |  |  |  |  |  |
| 3. Democrat threat | 2.57 | 1.21 | .23**  [.09, .36] | .31**  [.17, .43] | -- |  |  |  |  |
| 4. Independent/ undecided threat | 2.18 | 1.10 | .01  [-.13, .16] | .38**  [.25, .50] | .56**  [.45, .66] | -- |  |  |  |
| 5. Republican affiliation | 4.01 | 2.32 | .69**  [.60, .76] | -.43**  [-.54, -.30] | .24**  [.10, .37] | -.00  [-.15, .15] | -- |  |  |
| 6. Democrat affiliation | 4.62 | 2.29 | -.53**  [-.63, -.41] | .52**  [.41, .62] | -.05  [-.20, .09] | .15*  [.00 .29] | -.36**  [-.48, -.22] | -- |  |
| 7. Independent/ undecided affiliation | 4.78 | 2.77 | .07  [-.08, .22] | -.00  [-.15, .14] | .03  [-.12, .18] | .05  [-.10, .20] | -.05  [-.20, .10] | .04  [-.11, .19] | -- |

Note. **p*<.05; ***p*<.01. Numbers within brackets are the 95% confidence intervals. Higher values for political ideology indicate greater conservatism.

Supplemental Table 2. *Regression models predicting political affiliation (Republican, Democrat, undecided) threat perceptions in Experiments 1 & 2.*

|  | **Experiment 1** | | | **Experiment 2** | | |
| --- | --- | --- | --- | --- | --- | --- |
| *Predictors* | *Estimates* | *95% CI* | *p* | *Estimates* | *95% CI* | *p* |
| (Intercept) | 3.24 | 2.98 – 3.51 | **<.001** | 3.30 | 3.12 – 3.49 | **<.001** |
| Political Affiliation  [Democrat] | -0.29 | -0.66 – 0.08 | .129 | -0.73 | -1.00 – -0.47 | **<.001** |
| Political Affiliation  [undecided] | -0.83 | -1.20 – -0.45 | **<.001** | -1.12 | -1.39 – -0.86 | **<.001** |
| Perceiver Political Ideology | -0.86 | -1.12 – -0.59 | **<.001** | -0.86 | -1.05 – -0.68 | **<.001** |
| Political Affiliation [Democrat] *  Perceiver Political Ideology | 1.50 | 1.13 – 1.87 | **<.001** | 1.14 | .88 – 1.41 | **<.001** |
| Political Affiliation  [undecided] *  Perceiver Political Ideology | 0.86 | 0.48 – 1.12 | **<.001** | 0.88 | 0.61 – 1.15 | **<.001** |

Note. The Political Affiliation reference condition is Republican.

Supplemental Table 3. *Means (M), standard deviations (SD), and intercorrelations (r) between composite political ideology and other partisan experience measures in Experiment 1.*

| Measure | *M* | *SD* | 1 | 2 | 3 | 4 | 5 | 6 | 7 |
| --- | --- | --- | --- | --- | --- | --- | --- | --- | --- |
| 1. Perceiver political ideology | 4.79 | 1.91 | -- |  |  |  |  |  |  |
| 2. Republican:  percent of family | 49.51 | 35.51 | .37*  [.24, .49] | -- |  |  |  |  |  |
| 3. Democrat:  percent of family | 36.83 | 32.35 | -.43**  [-.54, -.30] | -.73**  [-.79, -.66] | -- |  |  |  |  |
| 4. Independent/ Undecided:  percent of family | 13.86 | 24.92 | .04  [-.11, .18] | -.47**  [-.58, -.35] | -.26**  [-.39, -.11] | -- |  |  |  |
| 5. Republican:  percent of friends | 33.54 | 23.91 | .34**  [.20, .46] | .40**  [.27, .51] | -.22**  [-.36, -.08] | -.27**  [-.40, -.13] | -- |  |  |
| 6. Democrat:  percent of friends | 48.33 | 25.62 | -.30**  [-.43, -.16] | -.10  [-.24, .05] | .31**  [.17, .43] | -.26**  [-.39, -.12] | -.50**  [-.60, -.38] | -- |  |
| 7. Independent/ Undecided:  percent of friends | 18.14 | 24.90 | -.02  [-.16, .13] | -.28**  [-.41, -.14] | -.10  [-.25, .04] | .53  [.41, .63] | -.45**  [-.56, -.32] | -.55**  [-.65, -.44] | -- |

Note. **p*<.05; ***p*<.01. *p*-values are unadjusted. Numbers within brackets are the 95% confidence intervals. Higher values for political ideology indicate greater conservatism.

Supplemental Table 4. *Means (M), standard deviations (SD), and intercorrelations (r) between political ideology, perceived threat, and political affiliation in Experiment 2.*

| Measure | *M* | *SD* | 1 | 2 | 3 | 4 | 5 | 6 | 7 |
| --- | --- | --- | --- | --- | --- | --- | --- | --- | --- |
| 1. Political ideology | 5.03 | 1.79 | -- |  |  |  |  |  |  |
| 2. Republican threat | 3.25 | 1.72 | -.50**  [-.64, -.33] | -- |  |  |  |  |  |
| 3. Democrat threat | 2.95 | 1.34 | .48**  [.31, .62] | .07  [-.14, .27] | -- |  |  |  |  |
| 4. Independent/ undecided threat | 2.42 | 1.17 | -.00  [-.21, .20] | .36**  [.17, .53] | .30**  [.11, .48] | -- |  |  |  |
| 5. Republican affiliation | 4.49 | 2.41 | .77**  [.67, .84] | -.55**  [-.68, -.40] | .51**  [.34, .65] | -.04  [-.24, .17] | -- |  |  |
| 6. Democrat affiliation | 4.47 | 2.38 | -.62**  [-.73, -.48] | .62**  [.47, .73] | -.40**  [-.56, -.21] | .17  [-.04, .36] | -.53**  [-.66, -.36] | -- |  |
| 7. Independent/ undecided affiliation | 4.64 | 2.44 | -.02  [-.23, .18] | .04  [-.17, .24] | .05  [-.16, .25] | -.14  [-.34, .07] | -.03  [-.23, .17] | .06  [-.15, .26] | -- |

*Note*. **p*<.05; ***p*<.01. Numbers within brackets are the 95% confidence intervals. Higher values for political ideology indicate greater conservatism.

Supplemental Table 5. *Means (M), standard deviations (SD), and intercorrelations (r) between composite political ideology and other partisan experience measures in Experiment 2.*

| Measure | *M* | *SD* | 1 | 2 | 3 | 4 | 5 | 6 | 7 |
| --- | --- | --- | --- | --- | --- | --- | --- | --- | --- |
| 1. Political ideology | 5.03 | 1.79 | -- |  |  |  |  |  |  |
| 2. Republican:  percent of family | 51.84 | 34.94 | .41*  [.23, .57] | -- |  |  |  |  |  |
| 3. Democrat:  percent of family | 32.14 | 30.60 | -.46**  [-.61, -.29] | -.67**  [-.77, -.54] | -- |  |  |  |  |
| 4. Independent/ Undecided:  percent of family | 4.47 | 2.38 | -.62**  [-.73, -.48] | -.43**  [-.58, -.25] | .58**  [.42, .70] | -- |  |  |  |
| 5. Republican:  percent of friends | 37.93 | 26.12 | .45**  [.27, .60] | .51**  [.35, .65] | -.36**  [-.52, -.16] | -.47**  [-.61, -.29] | -- |  |  |
| 6. Democrat:  percent of friends | 46.88 | 26.68 | -.45**  [-.60, -.27] | -.26*  [-.44, -.06] | .46**  [.29, .61] | .46**  [.28, .60] | -.68**  [-.77, -.55] | -- |  |
| 7. Independent/ Undecided:  percent of friends | 15.19 | 21.27 | .01  [-.19, .21] | -.30**  [-.47, -.10] | -.14  [-.34, .06] | -.00  [-.20, .20] | -.38**  [-.54, -.19] | -.42**  [-.58, -.24] | -- |

Note. **p*<.05; ***p*<.01. *p*-values are unadjusted. Numbers within brackets are the 95% confidence intervals. Higher values for political ideology indicate greater conservatism.

Supplemental Table 6. *Linear mixed effects model predicting evaluations in Experiment 2.*

| *Predictors* | *Estimates* | *95% CI* | *p* |
| --- | --- | --- | --- |
| (Intercept) | 3.70 | 3.55 – 3.84 | **<.001** |
| Label [Democrat] | 0.02 | -0.11 – 0.15 | .786 |
| Label [undecided] | 0.00 | -0.10 – 0.11 | .971 |
| Time [After Label] | -0.14 | -0.20– -0.08 | **<.001** |
| Political Ideology | -0.07 | -0.21 – 0.07 | .341 |
| Label [Democrat] *  Time [After Label] | 0.09 | 0.01 – 0.17 | **.030** |
| Label [undecided] *  Time [After Label] | 0.19 | 0.11 – 0.28 | **<.001** |
| Label [Democrat] *  Political Ideology | 0.04 | -0.09 – 0.17 | .537 |
| Label[undecided] *  Political Ideology | 0.01 | -0.09 – 0.12 | .829 |
| Time [After Label] *  Political Ideology | 0.49 | 0.43 – 0.55 | **<.001** |
| Label [Democrat] *  Time [After Label] *  Political Ideology | -0.82 | -0.90 – -0.73 | **<.001** |
| Label [undecided] *  Time [After Label] *  Political Ideology | -0.56 | -0.64 – -0.47 | **<.001** |

*Note*. The Label reference group is Republican.
