# Supplementary material for: Disclosing political partisanship polarizes first impressions of faces
Source: PLoS One. 2022 Nov 9;17(11):e0276400. doi: 10.1371/journal.pone.0276400 (PMC9645606; doi:10.1371/journal.pone.0276400)
Supplement: Attachment — Submitted filename: Response to Reviewers.docx [file pone.0276400.s002.docx]

**Responses to Individual Reviewer Concerns:**

**Reviewer 1**

*Comment 1:* The reviewer had a broad remaining concern that perceived threat should not be discussed as a mechanism for polarized face impressions based on disclosed partisanship because the manuscript provides no experimental evidence of it. The reviewer also notes that inconsistencies regarding perceived partisan threat both within- and across-experiments should be addressed in discussion sections and in the general discussion. The reviewer suggested a broader discussion of potential mechanisms for face impressions polarized by partisanship, especially in the general discussion.

*Action taken:* These were points well taken. We absolutely agree with the reviewer that causality must not be inferred when only correlational evidence for a relation can be presented. We have revised our manuscript in several ways to address this concern.

First, we have removed language speaking to causality throughout the manuscript except when we suggest that future work use experimental manipulations of, for example, perceived partisan threat to establish a causal link with polarized face impressions (e.g., p. 4, 34).

Second, we now discuss inconsistencies in perceived threat findings within- and across-experiments, being cautious to avoid redundancy. For example, we write in the Experiment 1 Discussion on p. 23, “Although threat ratings suggested that more conservative perceivers did not find Democrats as more threatening than Republicans, significant correlations emerged between perceiver ideology and partisan threat perceptions. What might have caused this inconsistency? One possibility may lie in the college-aged sample recruited for the experiment. College-aged students often show a bias to perceiving themselves as more conservative than they really are (Zell & Bernstein, 2014). If the students identifying themselves as more conservative were indeed more liberal than they realized, it would allow for the possibility of threat perceptions less extreme than those of the students identifying as more liberal. Indeed, these biased perceptions of one’s own partisanship are more pronounced for conservatives than for liberals (Zell & Bernstein, 2014).”

We also address these inconsistencies while considering new potential mechanisms in the General Discussion on p. 35 by writing, “Although ideology effects on face impressions were paralleled by partisan threat perception effects across experiments, it is worth considering why inconsistencies across experiments might emerge. One previously discussed possibility regarded college students self-reporting being more conservative than they are when ideology is more objectively assessed (Zell & Bernstein, 2014). Potential conflicts between self-reported and actual ideologies could lead to inconsistencies both within- and across-experiments. Speculatively, more objective ideology assessments could, in part, resolve inconsistencies. It could be that factors that are beyond the scope of the current work interfaced with perceived threat and ideology to relate to impressions. For example, people who have high actual (Whitt et al., 2021) or even imagined (Warner & Villamil, 2017) contact with opposing partisans have less affective polarization, findings that broadly reflect work on intergroup contact to reduce prejudice (Pettigrew, 1998). Future work may consider the extent to which relative partisan contact or isolation interfaces with perceived threat to affect face impressions separably or interactively.”

*Comment 2:* “Prior work supports disclosed partisanship as more generally polarizing face impression.”
(p. 4): The baseline for comparison is missing here (“more” than what?):

*Action taken:* We have rewritten the sentence for clarity. It now reads (p. 4), “Prior work supports that disclosed partisanship may polarize face impressions across contexts.”

*Comment 3:* I think partisan cues, and especially the labels used in this work, are not minimal cues as claimed on p. 6. They indeed seem very salient.

*Action taken:* We agree and have rewritten the sentence for clarity. It now reads (p. 6), “Such patterns would extend work showing favoritism and, sometimes, derogation, based on group membership (Brewer, 1999; Tajfel, 1970; Tajfel et al., 1971; Tajfel & Turner, 1979) from a romantic (Mallinas et al., 2018) to a more general context and show that simple partisan labels in the absence of other partisan information can powerfully affect impressions.

*Comment 4:* “Although people accurately detect political partisanship from faces…” (p. 7): this sentence reads a bit too deterministic; they "can" detect, or they "tend to" detect?

*Action taken:* We have rewritten the sentence to read (p. 8), “Although people can often detect political partisanship from faces (Rule & Ambady, 2010), disclosed group labels can override naturally occurring ones to elicit biases (Van Bavel et al., 2008).”

*Comment 5:* It seems odd to drop a participant’s data entirely for not reporting their age in Study 1. What is the rationale behind this decision? Were the results replicated when all participants were included in the analyses (at least this one participant who did not fail the attention and manipulation check)?

*Action taken:* We now report analyses from Experiment 1 that include the participant who did not report his/her age. None of the conclusions or statistical significance of the results changed.

*Comment 6:* How many task versions were there in total (including counterbalancing versions)? That would be nice to include in the main text (although one can probably figure it out by browsing the data files).

*Action taken:* We now clarify that there were three versions of Experiment 1 (p. 10) and three versions of Experiment 2 (p. 26).

*Comment 7:* This interpretation was not clear to me: “Main effects of Disclosed Label Veracity (reflecting fewer selected Democrats with accurate versus inaccurate labels) and Perceiver Political Ideology (reflected fewer selected Democrats with higher perceiver conservatism) emerged. Note that the Disclosed Label Veracity effect likely reflects the ideological skew of the sample” (p. 16). Checking the distribution reported in the supplementary materials, the sample seems to be a little bit skewed towards Democrats. But wouldn’t we expect the opposite of these results were due to this skewness (i.e., more selected Democrats with accurate labels)? Perhaps the authors can clarify that part a bit more in the text.

*Action taken:* We have clarified this interpretation in the Experiment 1 Results section on p. 16 by writing, “A main effect of Perceiver Political Ideology reflected fewer selected Democrats with higher perceiver conservatism. A main effect of Disclosed Label Veracity reflected fewer selected Democrats with accurate versus inaccurate labels. This pattern may seem surprising because given the liberal skew of the sample (see Supplemental Material), one might expect more accurately labeled Democrats because potential detections would match labels. Because differences between faces signal the likelihood of winning (Todorov et al., 2005), it could also be that inaccurate labels resulted in more “Democrats” with positively interpreted facial cues.”
